# High-spatial and colourimetric imaging of histone modifications in single senescent cells using plasmonic nanoprobes

**DOI:** 10.1038/s41467-021-26224-9

**Published:** 2021-10-08

**Authors:** Hyun Ji An, Yun Kim, Soojeong Chang, Hakchun Kim, Jihwan Song, Hyunsung Park, Inhee Choi

**Affiliations:** 1 Department of Life Science, University of Seoul, Seoul, 02504 Republic of Korea; 2 Department of Mechanical Engineering, Hanbat National University, Daejeon, 34158 Republic of Korea

**Keywords:** Surface plasmon resonance, Senescence, Predictive markers, Nanoparticles, Imaging techniques and agents

## Abstract

Histones are closely related to the state of chromatin, and epigenetic modification of their tail results in regulation in cells. Therefore, developing various analytical tools to map the changes in position and distribution of histone modifications is helpful in studying underlying mechanisms. Herein, we propose a high-spatial and colourimetric imaging method using plasmonic nanoparticles as probes to visualize heterochromatin histone markers in a single nucleus. We visualized the reorganization between repressive histone markers, H3K9me3 and H3K27me3, caused by oncogene-induced senescence based on the scattering colours and spectral shift of plasmonic nanoprobes to longer wavelengths using their distance-dependent coupling effect. The measured scattering profiles were correlated with the computation results simulating the scattering spectra according to the arrangements and distances among the plasmonic nanoprobes. The plasmonic nanoprobe-based high-spatial hyperspectral imaging provides an advanced way to study the dynamics of histone modifications for predicting the progression of diseases or senescence.

## Abstract (web summary)

The authors demonstrate a colourimetric imaging method using plasmonic nanoparticles for visualisation of heterochromatin histone markers. Based on the distance-dependent coupling effect, resulting in spectral shifts, they observe reorganisation of histone markers caused by oncogene-induced senescence.

## Introduction

Histone modifications are main causes of dramatic topological changes in chromatin^1,2^. Diverse combinatorial modifications of histones, named “histone code”, are determined by the balance between a variety of writer enzymes and eraser enzymes^3–5^. The information in the histone code is recognized by reader proteins which are involved in almost all events on chromatin such as transcription, repair, replication, and chromatin topology^5,6^. For example, the chromodomain of heterochromatin protein-1 (HP1), a reader protein recognizes and binds H3K9me3, the trimethylation of the 9th lysine residue of histone 3. After binding, HP1 oligomerizes itself and the oligomerization of HP1 makes chromatin structure more compact; thus, H3K9me3 is often found in heterochromatin, a condensed chromatin structure^7,8^. Interaction between HP1 and lamin A, a structural protein constituting the inner membrane of the nuclear envelope, causes H3K9me3 to be closely located to the inner membrane of the nuclear envelope. Through these mechanisms, H3K9me3 can determine the structure and three-dimensional location of the chromatin region. Recent findings that expand on other heterochromatin histone markers, such as H3K27me3, H3K56me3, H3K64me3, and H4K20me3, increase complexity in the relationship between histone code and chromatin topology^9–13^. The genome-wide analyses of mutations in cancers have identified tens of genes encoding in histones, DNA/histone modifying enzymes, erasing enzymes, and chromatin remodeling complex as cancer driver genes, suggesting that changes in histone code and chromatin structure are closely related in cancer development^14–23^. Accordingly visualizing the spatial distribution of histone modifications in a single cell level can allow for extended application to diagnose cancer or other disease-specific cellular phenotypes as well as senescence.

To develop a novel imaging method for analysing spatial distribution of histone codes using plasmonic nanoprobes, we used oncogene-induced senescent (OIS) cells as a model system because OIS process induced dramatic changes in heterochromatin distribution. Oncogenes such as *Ras*, *Raf*, and *Myc* induce cellular senescence if there are no additional mutations of tumor suppressor genes^24–26^. Immunostaining of OIS cells using an antibody specific for H3K9me3 revealed distinct clustered patterns of H3K9me3, named senescence-associated heterochromatin foci (SAHF)^27–29^. In contrast, in growing cells, H3K9me3 was detected as a diffused pattern inside the nuclei and was concentrated along the inner membrane of the nuclear envelope. During OIS process, spatial rearrangements of several histone modifications such as H3K9me3 and H3K27me3 and their reader proteins establish SAHF formation^30–32^. Therefore, the precise spatial arrangement of histone modifications in the nucleus can represent unique feature of epigenetic status of growing, aging, or tumor cells. However, conventional histone imaging methods using organic fluorescent dyes have limitations, such as photobleaching, poor resolution, and obligatory use of secondary antibodies.

To overcome the drawbacks of conventional organic fluorophores, nano-sized inorganic probes, including quantum dots and metallic nanoparticles, have drawn attention. Recently, plasmonic nanoparticles, such as gold nanoparticles (GNPs) and silver nanoparticles (SNPs), have been utilized in various imaging^33–35^ and sensing^36–39^ applications owing to their excellent optical properties, photostability, biocompatibility, and ease of synthesis and surface modification. The optical properties of plasmonic nanoparticles can be tuned by adjusting their shape, size, and composition. In particular, when two or more plasmonic nanoparticles are placed in close proximity, they exhibit the plasmonic coupling effect, which induces shifts of spectra, such as absorbance and scattering^40,41^. These spectral shifts are closely dependent on the interparticle distance, arrangement, and type of plasmonic nanoparticles. This effect has been frequently applied to study molecular interactions and distribution^42,43^. Despite the excellent optical properties of plasmonic nanoparticles, no attempt has been made to visualize histone modifications in a single nucleus until now.

**Fig. 1:**
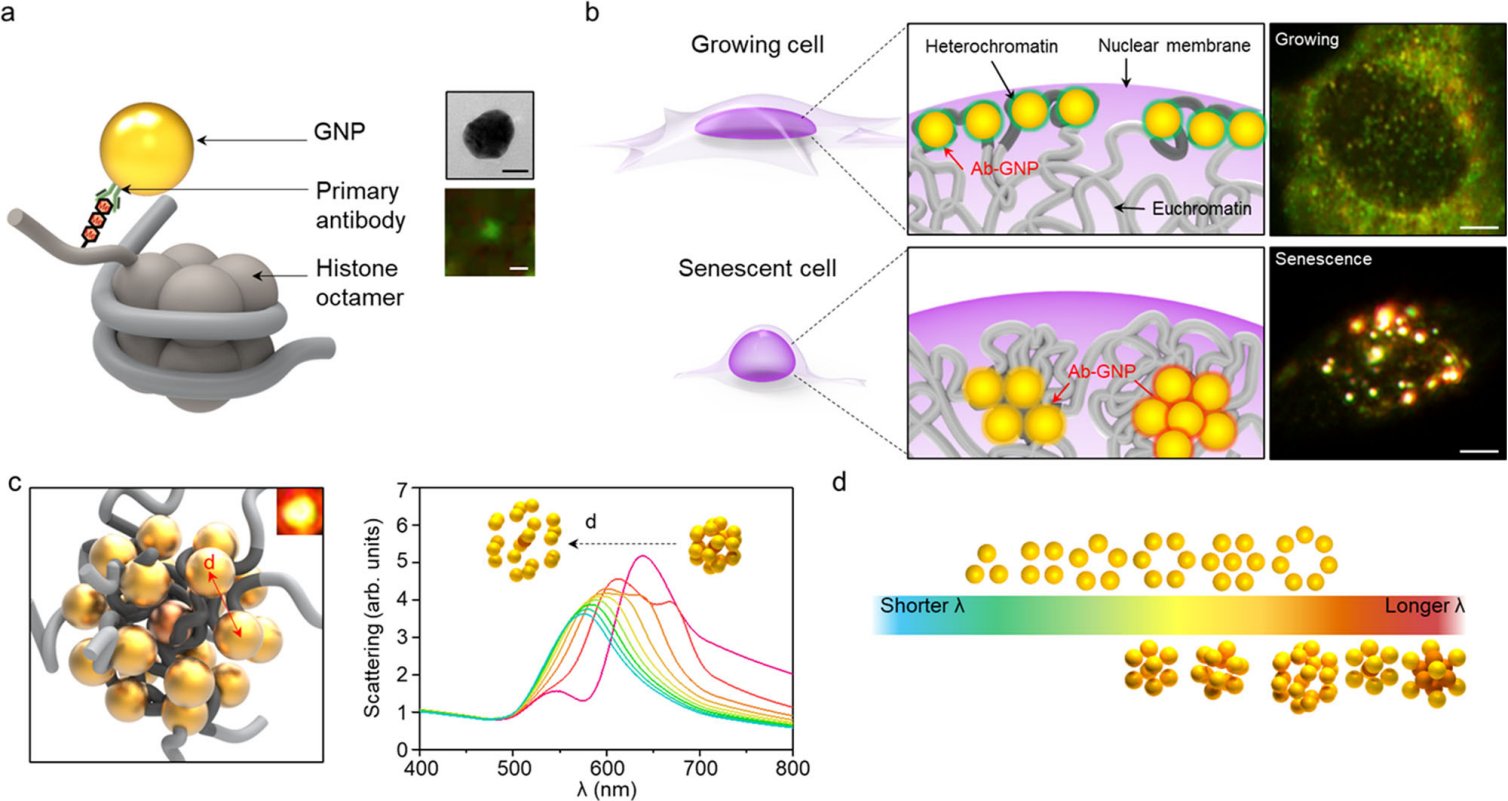
Schematic illustrations of histone imaging with plasmonic nanoprobes in a single cell. **a** Plasmonic nanoprobe conjugated with primary antibody of histone modification marker. Electron micrograph and dark-field scattering images of a single gold nanoparticle (GNP), images representative of five experiments. Black scale bar, 20 nm. White scale bar, 500 nm. **b** Representative dark-field scattering images of the plasmonic nanoprobes conjugated with antibody (Ab-GNP) targeting heterochromatin histone markers in a growing cell and a senescent cell. Images representative of five experiments. Scale bar, 10 µm. **c** An example of spatial arrangements of the plasmonic nanoprobes targeted to heterochromatic markers and simulated spectra of distance-dependent scattering of the probes. **d** Trends in the spectral peak (*λ*_max_) of simulated scattering according to the arrangement of plasmonic nanoprobes.

Herein, we utilize plasmonic nanoparticles conjugated with primary antibodies for heterochromatin-specific histone modifications, H3K9me3 and H3K27me3, to achieve high-spatial and colourimetric imaging of histone markers in a single nucleus. By virtue of the distance-dependent plasmonic coupling effect between nanoprobes, we observe the formation of SAHFs with dynamic color change of the probes assembled on the histone modification sites (Fig. 1a, b). Furthermore, we estimate their possible arrangements and distances from the scattering profiles of the nanoprobes labeled to the histone modifications interpreted with optical simulation (Fig. 1c, d). This indicates that multiple histone modification sites could be located within a nano-scaled distance in the nucleus of the senescent cell. The proposed method using spectral analyses of plasmonic probes will provide a novel way to visualize spatial changes in not only histone modifications but also other biomarkers at a single cell level for predicting the progression of diseases or senescence.

## Results

### Imaging of H3K9me3 reorganization during OIS

**Fig. 2:**
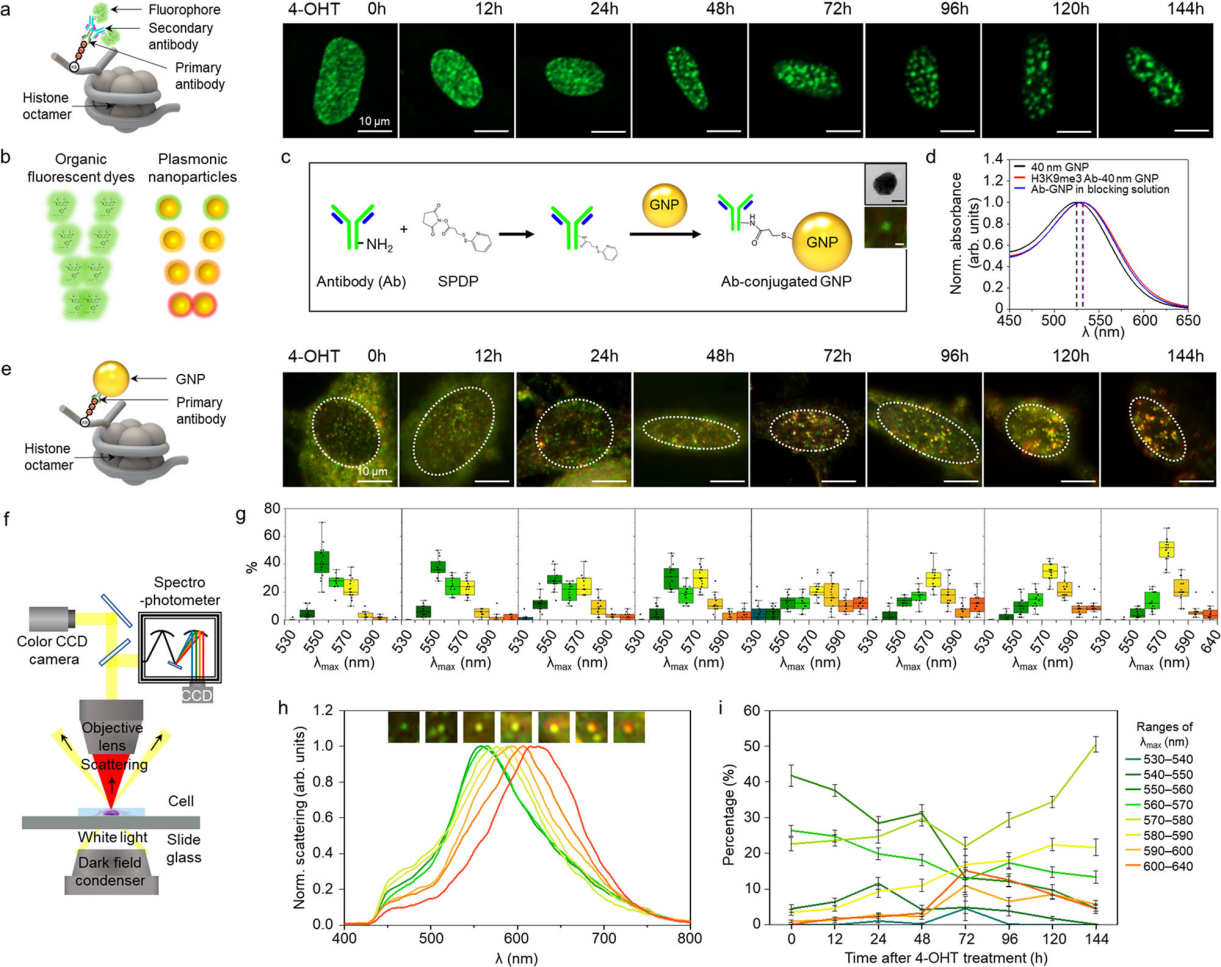
Single histone marker (H3K9me3) imaging in OIS cells. **a** Representative fluorescent image at each OIS time point using organic fluorophore conjugated with secondary antibody. Images, representing five experiments. Scale bar, 10 µm. **b** Comparison of the colors expressed by organic fluorophore and plasmonic nanoparticles according to the change in distance. **c** Conjugation strategy of primary antibody to 40 nm gold nanoparticle for preparing a plasmonic nanoprobe (Ab-GNP) with succinimidyl 6-[3′-(2-pyridyldithio)-propionamido] hexanoate (SPDP) linker. Images, representing five experiments. Black scale bar, 20 nm. White scale bar, 500 nm. **d** Change in absorbance before and after antibody conjugation to the gold nanoparticles. **e** Representative dark-field scattering images at each OIS time point. Images, representing five experiments. Scale bar, 10 µm. **f** Dark-field microscope setup for scattering imaging and spectral analysis of the plasmonic nanoprobes targeting histone marker. **g** *λ*_max_ distributions of the spectra of scattering spots (*n* = 50) in single cells (*n* = 15 cells examined over five independent experiments) during OIS. Box plots indicate median (middle line), mean (□), 25th, 75th percentile (box), and range within 1.5 interquartile range (IQR, whiskers) as well as each data point (●). **h** Changes in colors (inset) and spectra of the scattering spots induced by the targeted assembly of the probes. **i** Time-lapse measurement of the maximum scattering wavelength (*λ*_max_) for the plasmonic nanoprobes in the nuclei after 4-OHT treatment (*n* = 15 cells examined over five independent experiments). Data are represented as mean ± s.e.m.

We generated IMR90-ΔB-RAF:ER cells, human lung fibroblast IMR90 cells, which expressed a constitutively active form of B-Raf mutant (449–804 aa) fused with the ligand binding domain of human estrogen receptor (ER) (281–599 aa, single amino acid change at G525R) only upon treatment with ER ligands such as 4-OHT^44,45^. Treatment with 4-OHT stabilizes ΔB-RAF:ER fusion protein, which initiates OIS processes in IMR90-ΔB-RAF:ER cells. In the absence of 4-OHT, we visualized the diffused pattern of H3K9me3 in the nucleus of a growing cell using H3K9me3 primary antibody and a conventional fluorescent dye-conjugated secondary antibody (Fig. 2a). After 48 h of treatment with 4-OHT, H3K9me3 became clustered; then, after 144 h, SAHF were obviously revealed, suggesting that OIS spatially reorganized heterochromatin. However, due to blurring of the conventional organic fluorophores, the resolution of closely arranged H3K9me3 in SAHF was too limited to investigate the spatial reorganization of H3K9me3 during OIS. Moreover, H3K9me3 modifications tagged by the organic fluorescent dye showed an identical green emission color regardless of their distribution change, as illustrated in Fig. 2b. In contrast, when using plasmonic nanoparticles as imaging probes, owing to their distance-dependent coupling, as illustrated in Fig. 2b, we obtained diverse scattering colors as the distances among the targeted histone markers became closer during the OIS.

Figure 2c shows an example of the plasmonic nanoprobes conjugated with primary antibodies, which are specific for an individual histone modification. Considering the effective optical cross-section for visualization by scattering, 40 nm gold and silver nanoparticles (i.e., GNP and SNP) were selected for conjugation with H3K9me3 and H3K27me3 antibodies. The sulfosuccinimidyl 6-[3′-(2-pyridyldithio)-propionamido] hexanoate (Sulfo-LC-SPDP) linker was utilized in the antibody-nanoparticle conjugation process. Before SPDP-antibodies were conjugated to the plasmonic nanoparticles, HEPES buffer was used to prevent nanoparticle aggregation and antibody inactivation^46^. These antibody-conjugated plasmonic nanoprobes were dispersed and stored in blocking solution to avoid non-specific binding and undesired aggregation before use^47^. UV–Vis absorbance spectra showing no large peak shifts and no additional peak formation indicated that the antibody-conjugated plasmonic nanoprobes were prepared without aggregation (see Fig. 2d and Supplementary Fig. 1). To confirm the specificity of the plasmonic nanoprobes, senescent cells were treated with bare GNPs and GNPs conjugated with either H3K9me3 antibody or H3K27me3 antibody. A sharp scattering peak with high intensity was observed in the nucleus of the antibody-GNP-treated cells, while the scattering signal from the nucleus was negligible in the bare GNPs-treated cells (Supplementary Fig. 2). It was also validated that the plasmonic nanoprobes enter into the nucleus of the permeabilized cells by showing their scattering spots at the middle plane of the nucleus. We note that no nanoprobes are observed in the nucleus of the non-permeabilized cells (Supplementary Fig. 3).

To visualize reorganization of the repressive histone marker H3K9me3 during OIS, we treated IMR90-ΔB-RAF:ER cells with 4-OHT for the indicated period of time to trigger OIS, then immunostained these cells with the H3K9me3 antibody-conjugated GNPs (Ab-GNP) (Fig. 2e). The scattering images of individual nuclei tagged with plasmonic gold nanoprobes exhibited a progressive color change from green to yellow or orange during the OIS processes. The scattering colors and spectra of the plasmonic nanoprobes in the OIS cells were collected via dark-field microscopy combined with a spectrophotometer (Fig. 2f). Based on measurement of the scattering spectra at 50 random spots per cell and classification of the scattering peaks, the distribution of the scattering peaks dynamically changed during OIS (Fig. 2g). Representative scattering colors and spectra observed in the OIS cells are displayed in Fig. 2h. In order to visualize the redistribution of H3K9me3 during OIS, the scattering profiles were systemically analyzed with 10 nm wavelength intervals for the indicated time after 4-OHT treatment (Fig. 2i). In growing cells (0 h after 4-OHT treatment), most scattering spots showed peaks in the range of 530–560 nm, close to the scattering wavelength of the original Ab-GNP probe. As OIS progressed, the peaks in the wavelength range of 530–570 nm decreased; however, the peaks in the range of 570–640 nm increased prominently. Obvious SAHF formed at 144 h after 4-OHT treatment, the largest population of scattering peaks was observed in the 570 to 580 nm range. Scattering peaks at wavelengths greater than 600 nm (maximum 640 nm) were frequently observed as yellow and orange colors in the nuclei of senescent cells. These peak wavelength shifts resulted from strong plasmon coupling between gold nanoprobes, which occurred due to close assembly of the targeted H3K9me3 in SAHF during the OIS.

In growing cells, noticeable background scattering in the cytosol was observed at the same focal plane. However, this is attributable to scattering by cellular organelles, not by free plasmonic probes. As shown in Supplementary Fig. 4, intrinsic background scattering was also observed in the non-treated cells and enhanced by antibody treatment; however, the scattering profiles were distinguishable from those of the Ab-GNP-treated cells in terms of spectral peak intensity and bandwidth. Furthermore, it was confirmed that the plasmonic nanoprobes conjugated with different antibodies, which are H3K4me2 (usually located in nucleus) antibody and TGF-β (usually located in cytosol) antibody, show different scattering patterns and positions (Supplementary Fig. 5). When targeting H3K4me2, the nanoprobes were less aggregated in the senescent cells than the cases of targeting to H3K9me3. On the contrary, TGF-β antibody-conjugated nanoprobes were observed only in the cytosol not in the nucleus.

### Computational analysis of optical properties of arranged nanoprobes

**Fig. 3:**
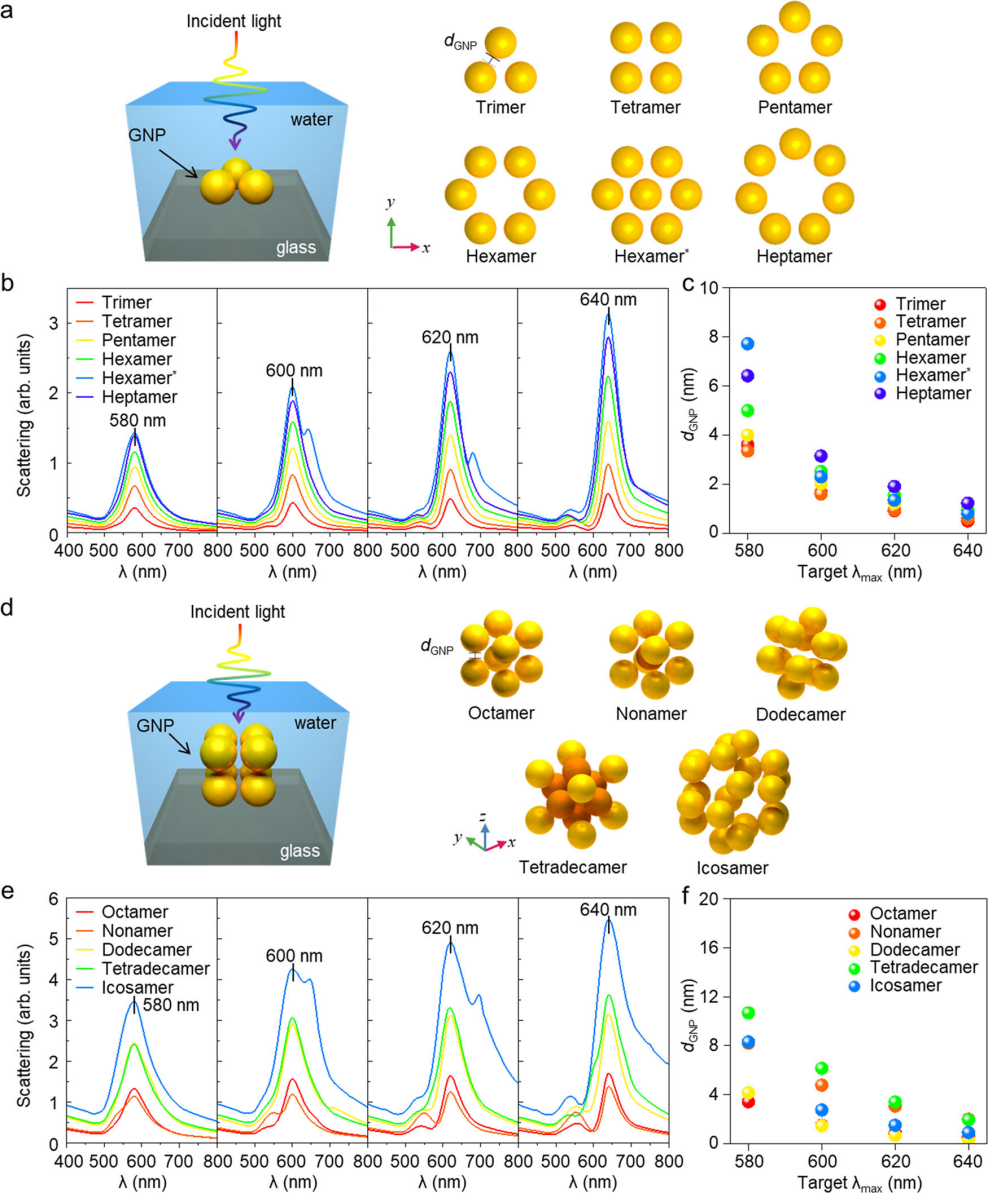
Computational simulations of the optical properties of arranged gold nanoprobes. **a** Various arrangements of GNPs in 2D. Domains in simulation and considered arrangements of GNPs, trimer to heptamer. **b** The scattering cross section of GNPs according to the target wavelength (*λ*_max_), 580–640 nm, left to right. **c** The expected interparticle distance of each 2D arrangement according to the *λ*_max_. **d** Various arrangements of GNPs in 3D. Domains in simulation and considered arrangements of GNP, octamer to icosamer. **e** The scattering cross section of GNPs according to the *λ*_max_, 580 to 640 nm, left to right. **f** The expected interparticle distance of each 3D arrangement according to the *λ*_max_.

To further understand the scattering signals from the Ab-GNPs according to the interparticle distance and their arrangement in OIS cells, computational simulations were carried out (see Fig. 3). Various assemblies, including 2-dimensional (2D) and 3-dimensional (3D), among the plasmonic nanoprobes were simulated considering different particle numbers (*N*_GNP_) and distances (*d*_GNP_). As shown in Fig. 3a, d, the optical simulation domain consists of the GNPs, which were placed on the glass and immersed in water, similar to mounting solution for imaging. The plane wave of the incident light propagates along the z-direction. In the case of 2D arrangements of GNPs, six kinds of typical structures were considered: trimer (*N*_GNP_ = 3), tetramer (*N*_GNP_ = 4), pentamer (*N*_GNP_ = 5), hexamer (*N*_GNP_ = 6), hexamer with a core (i.e., hexamer^*^, *N*_GNP_ = 7), and heptamer (*N*_GNP_ = 7), as shown in Fig. 3a. In the case of 3D arrangements of GNPs, five kinds of plausible structures were considered: octamer (*N*_GNP_ = 8), nonamer (*N*_GNP_ = 9), dodecamer (*N*_GNP_ = 12), tetradecamer (*N*_GNP_ = 14), and icosamer (*N*_GNP_ = 20) (Fig. 3d). For the octamer, dodecamer, and icosamer, the GNPs are placed at the vertices of such polyhedrons. For the nonamer and tetradecamer, the GNPs are internally added into the octamer. To investigate the effect of interparticle distance, *d*_GNP_, which was determined by the distance between a GNP and the closest particle, varied from 1 to 9 nm.

First, to characterize the optical properties of the GNP-based structures, simulations with various arrangements and interparticle distances were performed. The spectrum of the scattering cross section showed a red shift as the GNPs became closer and *N*_GNP_ increased, as shown in Supplementary Fig. 6. In order to correlate with the experimental results observed in Fig. 2g, cases showing key scattering peaks (580, 600, 620, and 640 nm) are displayed in Fig. 3b, e. The results showed that the intensity of the scattering spectra increased as *λ*_max_ increased and *N*_GNP_ increased in general. Most of the simulated spectra showed a single peak, but only hexamer^*^ in 2D arrangements and several cases in 3D arrangements showed distinct double peaks of the spectrum. In Supplementary Fig. 6, the double peak became clear as *d*_GNP_ decreased. A dip and two peaks originated from the different plasmonic resonance mode^48–50^. For instance, the nonamer shows a distinct double peak with the combination of two bright modes (enhanced scattering) and a dark mode (reduced scattering). Thanks to these diverse spectra according to the various arrangements, the GNPs can be an excellent probe for analyzing the distribution of histone modification.

The *d*_GNP_ of each arrangement according to *λ*_max_ is presented in Fig. 3c, f. When *λ*_max_ is 580 nm, the *d*_GNP_ between GNP structures is distributed in a broad range, while the difference in *d*_GNP_ according to GNP arrangements decreases as *λ*_max_ increases. This is because the spectral shift resulting from coupling between the plasmonic particles is exponentially proportional to the interparticle distance (*λ ∝* exp(-*d*_GNP_))^51–56^. Thus, the effect of the *d*_GNP_ becomes more dominant than the effect of *N*_GNP_ when strong plasmonic coupling appears, such as a high *λ*_max_. Namely, H3K9me3 might have a close distance between them at the site where high *λ*_max_ is measured. However, when the GNPs are located in relatively weak coupling conditions, the *d*_GNP_ is affected by the arrangement of the GNPs as well as the *N*_GNP_. In the case of 2D arrangements, the hexamer^*^ shows a slightly shorter *d*_GNP_ than hexamer when *λ*_max_ is 600, 620, and 640 nm (Supplementary Fig. 7a). In the case of 3D arrangements (Supplementary Fig. 7b), when the GNPs are internally added into the octamer (i.e., nonamer and tetradecamer), the *d*_GNP_ is farther than those of the other structures with a greater number of GNPs (i.e., dodecamer and icosamer, respectively). This is because the GNPs are arranged like an s-polarization^50^ when the GNPs are internally added into the cubic structure, meaning the GNPs are aligned parallel to the incident light and so induce stronger plasmonic coupling.

Considering these simulated results, we estimated the distance between the tagged histone markers H3K9me3 with the numerical results of *d*_GNP_, presuming that all Ab-GNPs attached in the same direction (see Supplementary Fig. 8). The *d*_GNP_ and the diameter of the GNPs (*D* **=** 40 nm) were considered in the estimation (i.e., *d*_histone markers_ **=** *d*_GNP_ + 40 nm). In the experimental results, the highest percentage of scattering peaks was observed at 580 nm in OIS cells (144 h after 4-OHT, see Fig. 2g). This observation implies that multiple H3K9me3 markers tagged in most of the spot areas in the OIS cells are distributed within an average distance of 43.4–50.7 nm, assuming that H3K9me3 is arranged in simple 2D and 3D configurations as we simulated. Likewise, the observation that the maximum peaks of several scattering spots are around 640 nm in senescent cells indicates that the tagged H3K9me3 is distributed within a shorter distance of 40.3–42.0 nm at those spots.

### Simultaneous imaging of H3K9me3 and H3K27me3 in OIS cells

**Fig. 4:**
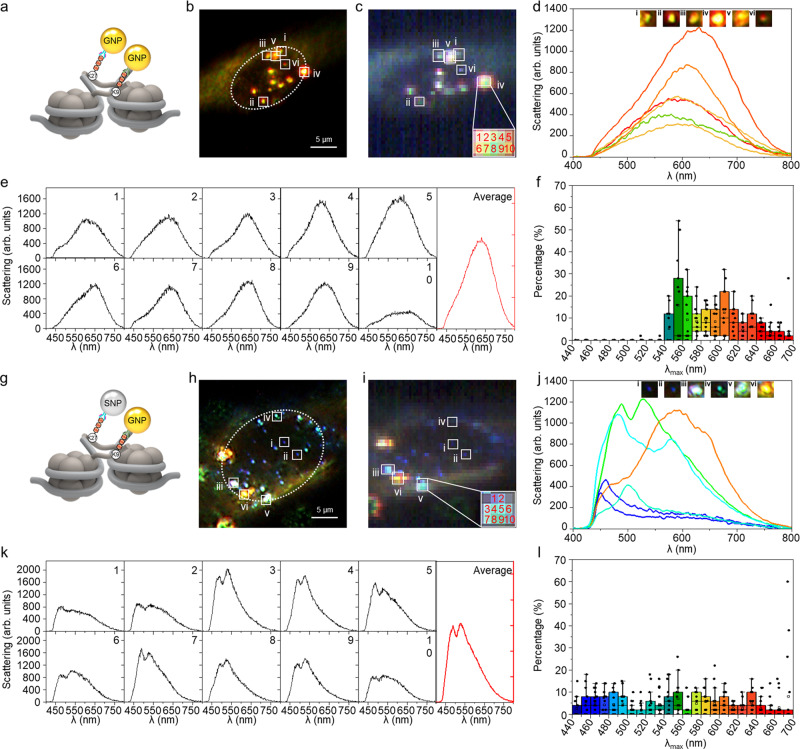
Dual histone markers imaging of H3K9me3 and H3K27me3 in OIS 144 h cells. **a**–**f** Analysis of the OIS cell using only GNPs for two different histone markers. **a** Dual targeting scheme using same GNPs conjugated with different antibodies for H3K9me3 and H3K27me3. **b** Representative dark-field scattering image of five experiments. Scale bar, 5 µm. **c** Hyperspectral imaging of the nanoprobes assembled in a single nucleus. In the hyperspectral image, inset grids indicate the occupied pixels by the single scattering spots. **d** Representative scattering colors and spectra observed in the OIS cell. **e** Representative scattering spectra measured from the observed scattering spots. Individual spectrum collected from each pixel in the single scattering spot is shown in black. An average of the collected spectra is shown in red. **f** *λ*_max_ distribution of the scattering spectra of the observed spots (*n* = 50) in each OIS time point cell (*n* = 15 cells examined over five independent experiments). Box plots indicate median (middle line), mean (□), 25th, 75th percentile (box), and range within 1.5 IQR (whiskers) as well as each data point (●). **g**–**l** Analysis of the OIS cell using GNPs and SNPs for respectively targeting two histone markers. **g** Dual targeting scheme using GNP for H3K9me3 and SNP for H3K27me3. **h** Representative dark-field scattering image of five experiments. Scale bar, 5 µm. **i** Hyperspectral imaging of the nanoprobes assembled in a single nucleus. **j** Representative scattering colors and spectra observed in the OIS cell. **k** Multiple spectra (black) collected from the pixels occupied by single scattering spots and their average spectrum (red). **l** *λ*_max_ distribution of the scattering spectra of the observed spots (*n* = 50) in each OIS time point cell (*n* = 15 cells examined over 5 independent experiments). Box plots indicate median (middle line), mean (□), 25th, 75th percentile (box), and range within 1.5 IQR (whiskers) as well as each data point (●).

Further analyses of the structure of the SAHF were performed by simultaneously targeting two histone modifications, H3K9me3 and H3K27me3, which are reported to be found in the core and periphery of SAHF, respectively^28,57,58^. We used two different combinations of plasmonic nanoparticles conjugated to either H3K9me3 or H3K27me3 antibodies. First, we conjugated GNPs to both H3K9me3 and H3K27me3 antibodies (Fig. 4a). Second, we conjugated GNP to H3K9me3 antibody and SNP to H3K27me3 antibody (Fig. 4g). In both cases, we frequently observed larger scattering spots (Fig. 4b, c, h, i) than in the case of single histone modification targeting (see Fig. 2e). This is due to the fact that H3K9me3 and H3K27me3 constitute compact SAHF^28,57,58^, and thus the number of plasmonic probes targeted to the histone markers in the SAHF increased. Therefore, to collect the scattering signal from each spot, spectra were collected from pixels that occupied a single SAHF and averaged (Fig. 4e, k and Supplementary Fig. 9). Compared to single targeting of H3K9me3, simultaneous targeting of both H3K9me3 and H3K27me3 showed drastic changes in scattering color and intensity (Fig. 4d, j), which can be attributed to stronger plasmonic coupling among the tagged probes coexisting in the scattering spots. Moreover, we observed wider red-shifts even at 700 nm when targeting H3K9me3 and H3K27me3 reorganized during OIS (Fig. 4f, l) compared to single histone marker imaging (i.e., H3K9me3 only; see Fig. 2g).

Notably, when using different plasmonic nanoprobes for targeting H3K9me3 and H3K27me3, that is, with 40 nm SNP and 40 nm GNP, the collected scattering profiles were diverse and the peak shift range broadened. To investigate the optical characteristics of the heterogeneous plasmonic structures consisting of GNPs and SNPs, spectral simulations for probable 2D and 3D assemblies were carried out by varying the interparticle distance (Supplementary Fig. 10). Simulation results also showed dramatic changes in scattering properties according to the arrangement and interparticle distance of the nanoprobes.

### Correlative analyses of experimental and simulated results

**Fig. 5:**
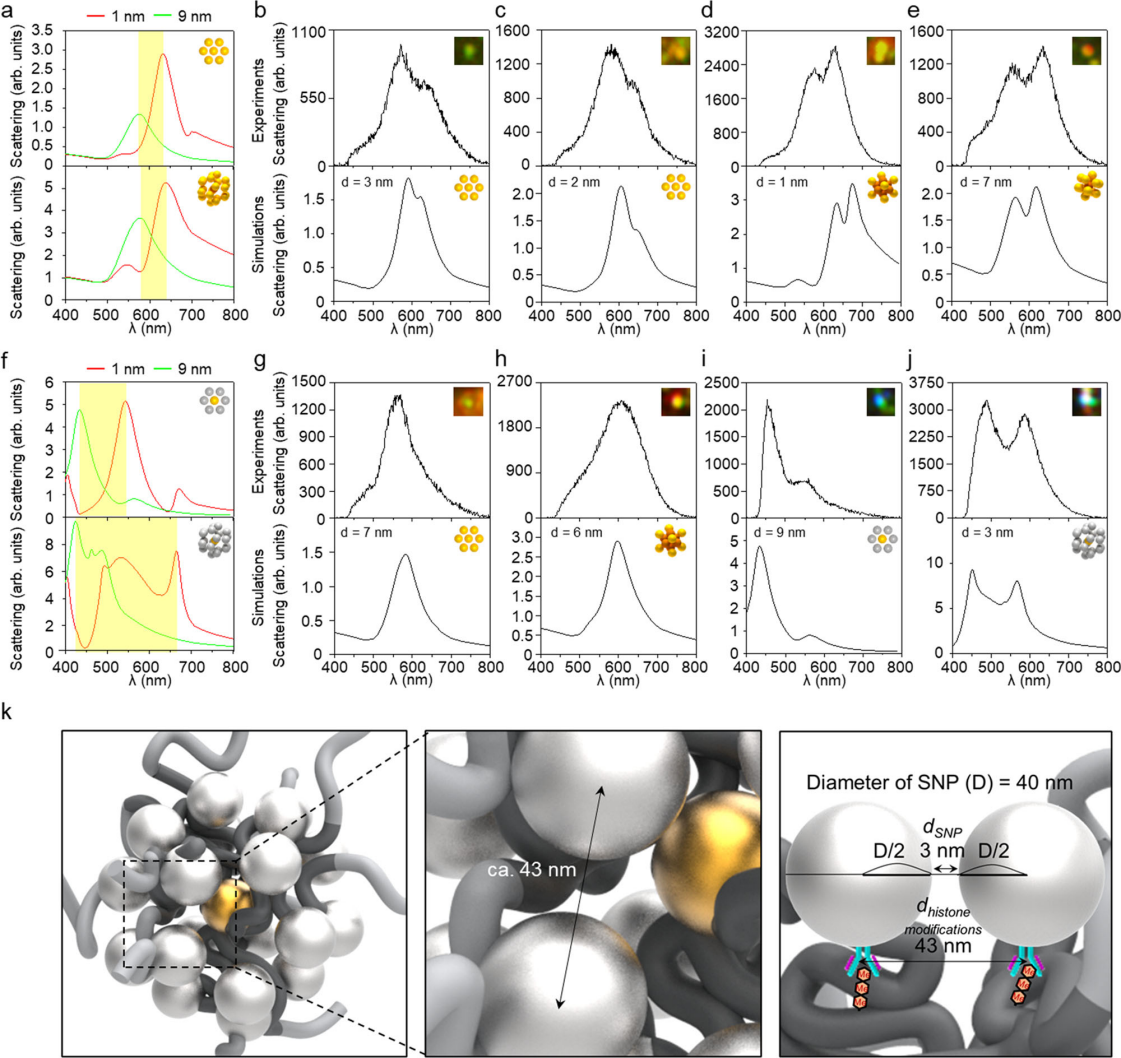
Correlative analysis of the collected scattering spectra of the plasmonic nanoprobes with computational analyses for plausible arrangements. **a** Spectral simulation for the representative 2D and 3D arrangements composed of only GNPs. Yellow boxes indicate the wavelength range of the spectral peaks for the assemblies with interparticle distance from 1 to 9 nm. **b**–**e** Correlation sets of the measured scattering spectra with simulation of the possible 2D and 3D arrangements of the GNPs targeting H3K9me3. **f** Spectral simulation for the representative 2D and 3D arrangements composed of GNP (core) and SNPs (surroundings). **g**–**j** Correlation sets of the measured scattering spectra with simulation of the possible 2D and 3D arrangements of the GNP and SNPs targeting H3K9me3 and H3K27me3, respectively. **k** An example of the distance estimated between histone markers on the basis of the experimental and numerical results of icosamer shown in **j**.

When compared with the GNPs/GNP-coupled structure (Fig. 5a), the SNPs/GNP-coupled structure (Fig. 5f) shows a wider range of shifts for the scattering spectrum when the interparticle distance varies. Owing to the higher gradient of the refractive index of silver compared to gold, the optical property of silver changes more dynamically with respect to the incident wavelength^59,60^. Furthermore, the SNPs/GNP-coupled 3D structure shows distinctive multiple peaks, as shown in Fig. 5f (lower portion). These distinctive spectra are derived from the bimetallic plasmonic interaction between the SNPs and the GNP^61^. These broad and distinctive spectra from SNPs/GNP-coupled structure can be useful for analyzing the distribution of two different histone modifications. In other words, it implies that the distribution of histone markers where different nanoprobes are attached can be more precisely analyzed than using a single type of nanoprobe.

To predict the distribution of individual histone modifications, we correlated the experimental data with the numerical simulation results, based on the optical characteristics of the plasmonic nanoprobes with various interparticle distances, arrangements, and compositions. Figure 5b–e shows the scattering spectra for the GNPs targeting only H3K9me3. The dark-field scattering images in the experimental results indicate the sites where the scattering spectra were obtained, and the inset images in the simulation results indicate the structures considered in the computation. The scattering spectra of the selected spots were compared with the simulated scattering spectra for 2D or 3D arrangements of nanoparticles. The displayed results were selected with the correspondences based on the wavelength of the peaks and the shape of the scattering spectra. For example, in the case of Fig. 5b, the experimental data shows *λ*_max_ around 570 nm and the second peak at 630 nm, of which the optical property is similar to that of the hexamer^*^ with an interparticle distance of 3.0 nm, showing a *λ*_max_ of approximately 570 nm and a second peak at about 630 nm. Figure 5c–e also shows the spectral similarity between the experimental and numerical results as well as the *λ*_max_. Based on the wavelength of the peaks and the shape of the scattering spectra, it is regarded that the targeted histone modifications are likely to be distributed as a hexamer shape with a core, as shown in Fig. 5b. In the same manner, several spatial arrangements of nanoprobes with interparticle distances of around 2.0, 1.0, and 7.0 nm can be predicted in Fig. 5c–e. This implies that multiple H3K9me3 markers were detected within 41.0–47.0 nm ranges in the observed scattering spots.

Figure 5g–j shows the scattering spectra for the targeting of two types of histone markers (H3K9me3 and H3K27me3). When it was targeted with a single nanoprobe (i.e., GNPs for both markers; Fig. 5g, h), an increase in the scattering intensity and a subtle spectral shift was observed in the majority of experimental results. Likewise, most of the simulation results show the change in scattering peak and intensity even though the structural configuration changes from 2D to 3D (see also Supplementary Fig. 6). For the observed spots, the distances between two markers tagged with the nanoprobes were estimated as 46.0 to 47.0 nm. This estimation suggests that the targeted histone modifications, H3K9me3 and H3K27me3, might be located in different nucleosomes, considering the size of each nucleosome (around 11–13 nm)^62,63^.

When dual nanoprobes (i.e., GNP and SNP) were used together (Fig. 5i, j), a distinctive difference appeared. According to the site where the scattering spectra were obtained experimentally, the spectral features varied from a single dominant peak (Fig. 5i) to strong double peaks (Fig. 5j). This significant difference was also observed in the simulation results. From dual targeting with two different nanoprobes, more abundant estimation could be possible with more diverse spectra. The histone modifications in Fig. 5i, j can be regarded as the shape of a 2D hexamer^*^ and a 3D icosamer with central GNP, whose distances are 9.0 nm and 3.0 nm, respectively. Based on the above estimation, it can be inferred that the histone modifications tagged with the nanoprobes were placed within 43.0 nm to 49.0 nm in the nucleus, as shown in Fig. 5k. In addition, we identified that targeted histone modifications are likely to be positioned three-dimensionally, based on spectral features collected from dual targeting imaging (see Supplementary Fig. 11). Accordingly, distinctive and diverse scattering spectra that are obtained by using two types of nanoprobes can be very helpful in understanding the spatial distribution of histone modifications in the nucleus.

Considering the diameter of the nanoprobes used (40 nm) and the size of nucleosomes (about 11–13 nm), it is impossible to detect multiple histone modifications in a single nucleosome as well as to target all histone markers in the nucleus. However, we would like to note that the proposed nanoprobe (i.e., antibody-conjugated plasmonic nanoparticles) specific for histone markers can successfully chase dynamic changes in the distribution of the targeted histone markers during OIS.

## Discussion

In summary, we have demonstrated the distribution of heterochromatin-specific histone modifications in a single nucleus using plasmonic nanoparticle-based high-spatial and colourimetric imaging methods. The distribution of the repressive histone markers, H3K9me3 and H3K27me3, was visualized via dark-field microscopy by targeting with plasmonic nanoprobes. As the distance between heterochromatin histone markers became closer with cellular senescence, the scattering colors and spectra of the plasmonic nanoprobes shifted to longer wavelengths, owing to the unique coupling effect of the plasmonic probes. This is further supported by simulation of the scattering profiles considering the particle numbers, interparticle distance, and spatial arrangement among plasmonic nanoprobes. Based on the correlation of the experimental and numerical scattering profiles, the spatial distribution of the targeted histone markers can be understood, and interparticle distance targeting histone markers can be estimated. In addition, further spectral interpretation of the three-dimensional distribution of H3K9me3 and H3K27me3 was accomplished by employing different types of plasmonic nanoparticles. Resultingly, we demonstrated plasmon-based colourimetric imaging to understand the spatial distribution and distance between histone modifications in an intact nucleus of a OIS cell for the first time. Although our present trial has limitations for targeting all histone markers and estimating distances below the probe size, this study could be further elaborated by using smaller plasmonic nanoparticles possessing enhanced scattering properties to obtain more detailed information for all of the modification sites. We anticipate that the proposed analytical technique combined with high-spatial imaging and spectral simulation will provide a new route to study cellular molecules, such as modified histones, and cellular states, and eventually lead to a new approach to diagnose and monitor the progression of diseases or cellular senescence.

To diagnose the pathological status of cells with the imaging of histone modifications, quantitatively sufficient, qualitatively information-intensive, and high-resolution images are required. Indeed, multi-omics approaches including histone ChIP-seq, transcriptome and mass spectrometry (MS)-based proteomics have produced enormous amounts of big-data to study the relationship between the histone modifications and the cellular functions^14,64^. MS-based middle-down and bottom-up proteomics discovered a variety of histone modifications throughout H2A, H2B, H3, and H4 isoforms. Considering all combinations, H3 can have more than 10^24^ kinds of different modifications, suggesting that the cell-specific combination patterns of histone modifications can represent information-intensive biomarkers. Proteomics analyses have been extensively attempted to assess tumor-specific combinations of histone modifications^64–66^. For example, H3K9me2/K14ac, H3K9me1/K14ac, and H3K9ac/K14ac were found less in triple-negative breast cancers than normal tissues^66^; H3K27me3/K36me1 level was found to be increased while H4K5ac/K8ac/K12ac/K16ac decreased in several cancer cell lines compared with embryonic stem cells^65^. Thus, if the cancer-type specific combinations of histone modifications in one histone molecule are identified with the above findings, our plasmonic nanoparticle-based imaging method could be further applicable for a cancer-type specific diagnosis by detecting specific combinatorial histone modifications.

## Methods

### Materials

Dulbecco’s modified Eagle’s medium (DMEM) and penicillin/streptomycin (PenStrep) were purchased from Gibco–Life Technologies (Carlsbad, CA, USA). Fetal bovine serum (FBS) was purchased from MP Biomedicals (Irvine, CA, USA). Bovine serum albumin (BSA), phosphate buffered saline (PBS), and 40 nm silver nanoparticles (SNPs) were purchased from Sigma-Aldrich (St Louis, MO, USA). The 40 nm gold nanoparticles (GNPs) were purchased from BBI Solutions (Crumlin, UK). Anti-Histone H3 (tri methyl K9) antibody and Anti-Histone H3 (di methyl K4) antibody were purchased from Abcam (Cambridge, MA, USA). Anti-Histone H3 (tri methyl K27) antibody was purchased from Millipore (MA, USA) and TGF-β1 polyclonal antibody from Biorbyt (Cambridge, UK). Goat anti-Rabbit IgG (H + L) cross-adsorbed secondary antibody, Alexa Fluor 488 was purchased from Invitrogen (MA, USA).

### Conjugation of plasmonic nanoprobes with antibodies

Bioconjugation of antibodies to plasmonic nanoparticles was carried out with the heterobifunctional crosslinker sulfosuccinimidyl 6-[3′-(2-pyridyldithio)-propionamido] hexanoate (Sulfo-LC-SPDP, 21650, Thermo)^67,68^. A total of 100 µM sulfo-LC-SPDP was prepared in ultrapure water immediately before use. Then, 25 µL of sulfo-LC-SPDP were added to 10 μg IgG dissolved in 0.5 mL of PBS-EDTA (100 mM sodium phosphate, 150 mM NaCl, 1 mM EDTA, 0.02% sodium azide, pH 7.5) and incubated for 30 min at room temperature (RT). Conjugated antibodies were exchanged with 40 mM HEPES buffer using a 10 K MWCO centrifugal filter (Millipore). Plasmonic nanoparticles (40 nm GNPs and 40 nm SNPs) were centrifuged (3000×*g*, 15 min) and resuspended in 40 mM HEPES buffer before antibody conjugation. Plasmonic nanoparticles and antibodies were mixed at a 1:250 molar ratio and incubated overnight at 4 °C.

### Construction of retrovirus expression vector and retrovirus production

The ΔB-Raf:ER fusion protein consists of a protein kinase domain of mouse B-Raf (449–804 aa) and a mutant form of the hormone-binding domain of the mouse estrogen receptor (281–599 aa, single amino acid change from glycine to arginine at 525) that has been engineered to be nonresponsive to β-estradiol but retains responsiveness to 4-hydroxy-tamoxifen (4-OHT)^29^. A 1 mM stock of 4-OHT was prepared in ethanol and used at a concentration of 100 nM.

### OIS cell culture

Human lung fibroblast IMR-90 cells were obtained from the ATCC (#CCL-186). IMR90 cells were transduced with a retrovirus encoding ΔB-Raf:ER. Cells were cultured in Eagle’s Minimum Essential Medium (MEM; #10-009-CV, Corning) supplemented with 10% fetal bovine serum (FBS), 100 IU/mL penicillin, and 100 μg/mL streptomycin. All cells were incubated under humidified air containing 5% CO_2_ at 37 °C.

### Cell incubation with plasmonic nanoprobes

Before treatment of the OIS cells, plasmonic nanoprobes were diluted in 0.1% BSA blocking solution. The OIS cells were fixed with 4% paraformaldehyde (PFA) for 15 min and washed with PBS at RT. Permeabilization was performed using 0.4% Triton X-100 in PBS for 10 min and 5 min washing, three times, with PBST (0.05% Tween-20 in PBS). After blocking the cells with 5% BSA in PBST for 1 h, plasmonic nanoprobes were treated and incubated overnight at 4 °C.

### Fluorescent imaging of OIS cell using organic fluorophore

The OIS cells were fixed with 4% paraformaldehyde (PFA) for 15 min and washed with PBS at RT. 0.4% Triton X-100 in PBS was treated for 10 min to permeabilize the cells and washed with PBST 5 min for three times. After blocking the cells with 5% BSA in PBST for 1 h, 1:250 diluted primary antibodies in blocking solution were treated to the cells overnight. Primary antibodies were washed ten times using PBS for 2 min each. Alexa 488 conjugated secondary antibodies diluted at a 1:500 ratio in the blocking solution were treated to the cells and incubated at RT for 1 h. After washing for ten times for 2 min each, the cells were preserved in a mounting solution.

### Single OIS cell imaging via dark-field microscopy

Imaging of histone codes targeted by plasmonic nanoprobes and measuring of the scattering spectra in various OIS time cells was performed using a dark-field microscope (Olympus BX43, Tokyo, Japan) equipped with a hyperspectral imaging spectrophotometer (CytoViva, Auburn, AL, USA). A 40× objective lens was used for imaging, and the integration time for collecting the scattering spectra was 0.3 s.

### Computational analyses of scattering spectra of arranged nanoprobes

To evaluate the optical behaviors of the plasmonic nanoparticles according to their distance, numbers, and arrangement, wave optics simulations were performed. Plane electromagnetic waves with wavelengths of 400–800 nm were applied. All simulations were carried out using commercial software (COMSOL Multiphysics 5.4).

### Reporting summary

Further information on research design is available in the Nature Research Reporting Summary linked to this article.

## Supplementary information

Supplementary Information

Reporting Summary

## Data Availability

Data that support the findings of this study are available in Figshare with the identifier 10.6084/m9.figshare.15169914. Source data are provided with this paper.

## Source data

Source Data
