# Supplementary material for: High-spatial and colourimetric imaging of histone modifications in single senescent cells using plasmonic nanoprobes
Source: Nat Commun. 2021 Oct 8;12:5899. doi: 10.1038/s41467-021-26224-9 (PMC8501099; doi:10.1038/s41467-021-26224-9)
Supplement: Supplementary file 1 — Supplementary Information [file 41467_2021_26224_MOESM1_ESM.pdf]

Supplementary Information

High-Spatial and Colourimetric Imaging of Histone Modifications in Single Senescent Cells Using Plasmonic Nanoprobes

Hyun Ji An[1, †], Yun Kim[2, †], Soojeong Chang[1, †], Hakchun Kim[1], Jihwan Song[2*], Hyunsung Park[1*], and Inhee Choi[1*]

[1]Department of Life Science, University of Seoul, Seoul 02504, Republic of Korea

[2]Department of Mechanical Engineering, Hanbat National University, Daejeon 34158, Republic of Korea

†These authors contributed equally.

Corresponding Authors: inheechoi1@uos.ac.kr; hspark@uos.ac.kr; jsong@hanbat.ac.kr

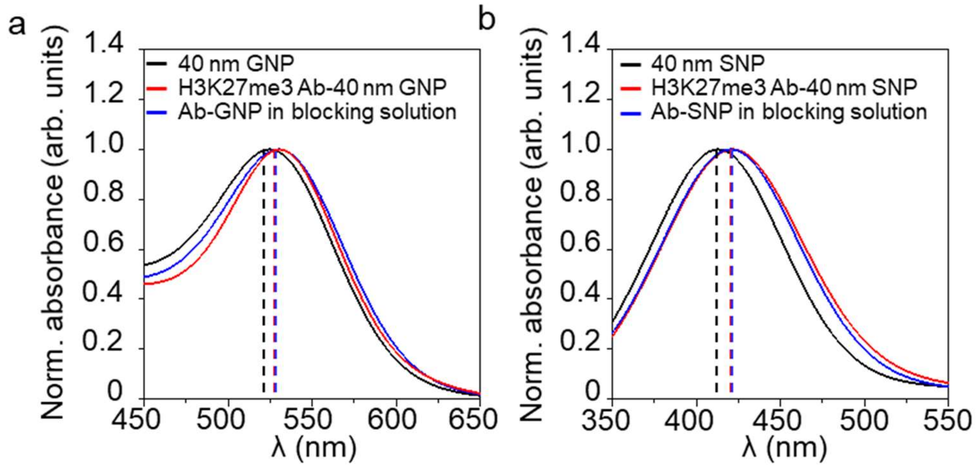

Supplementary Fig. 1 Absorbance peak shift of conjugated plasmonic nanoprobes. **a** Absorbance spectra for the 40 nm GNPs conjugated with H3K27me3 antibodies (Ab-GNPs), and the Ab-GNPs in blocking solution. The plasmonic peak of the 40 nm GNPs is 525 nm (black). The peak of the Ab-GNPs is observed at 530 nm (red) and that of the Ab-GNPs in blocking solution is observed at 531 nm (blue). **b** Absorbance spectra for the 40 nm SNP conjugated with H3K27me3 antibodies (Ab-SNPs) and the Ab-SNPs in blocking solution. The plasmonic peak of the 40 nm SNPs is 413 nm (black). The peak of the Ab-SNPs is observed at 420 nm (red) and that of the Ab-SNPs in blocking solution is observed at around 422 nm (blue).

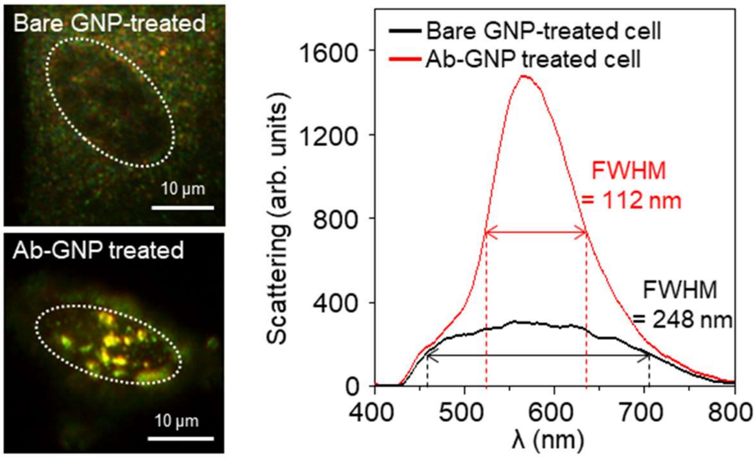

Supplementary Fig. 2 Identification of the targeting ability of the nanoprobes. Scale bar, 10 μm. Dark-field scattering images and scattering spectra of the bare GNP and the H3K9me3 Ab-GNP treated cells. In the scattering images, the dotted line marks the boundary of the nucleus. In the spectra, full width half maximum (FWHM) is indicated for comparison.

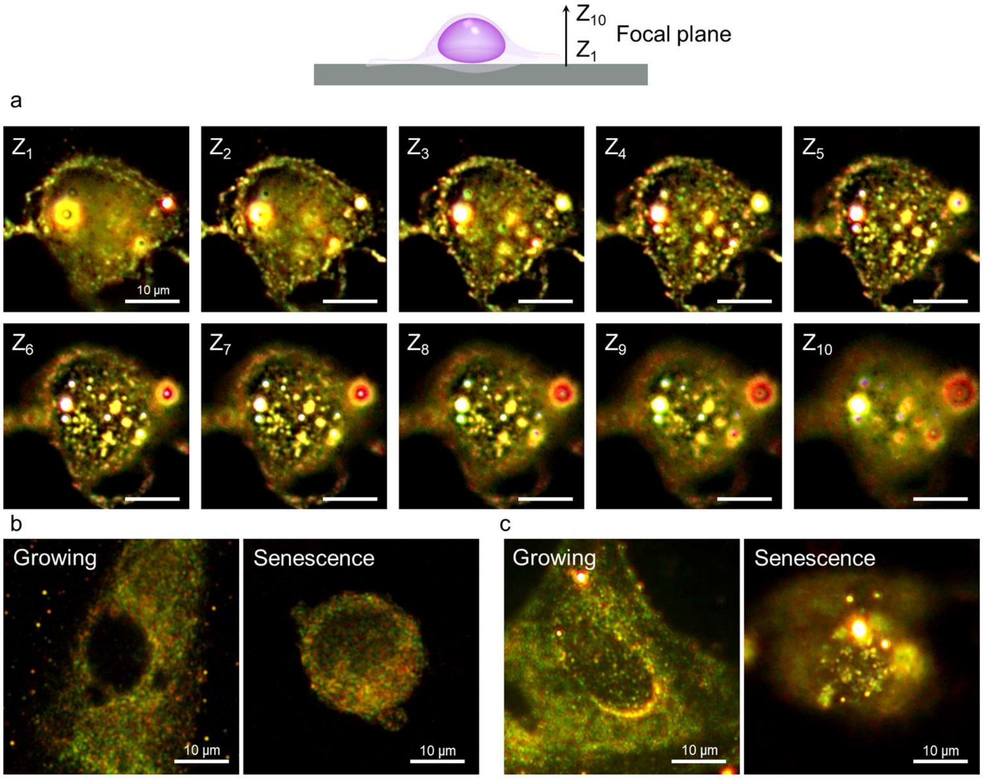

Supplementary Fig. 3 a Representative z-stack dark-field scattering images of H3K9me3 targeting Ab-GNPs treated OIS 144h cells. Scale bar, 10 µm. **b** Dark-field images of H3K9me3 targeting Ab-GNPs treated non-permeabilized growing cells (0 h after 4-OHT treatment) and senescent cells (144 h after 4-OHT treatment) cells. Scale bar, 10 µm. **c** Dark-field images of H3K9me3 targeting Ab-GNPs treated permeabilized growing and senescent cells. Scale bar, 10 µm. All images represent 4 experiments.

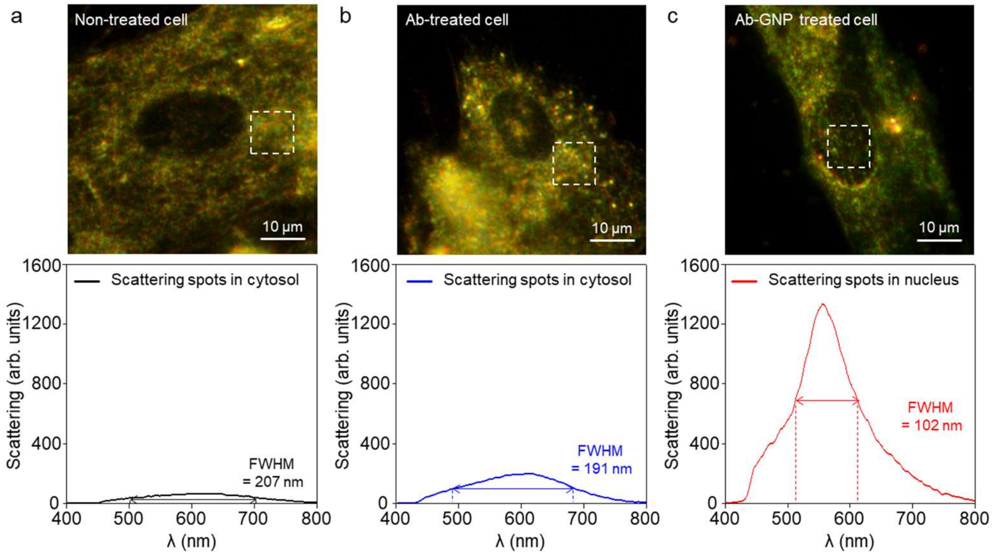

Supplementary Fig. 4 Comparison of background scattering from the 0 h 4-OHT treated cells with the scattering from the plasmonic nanoprobes. Dark-field scattering images of cells and corresponding scattering spectra obtained from each conditioned cell. Scale bar, 10 μm. **a** Non-treated cell. **b** H3K9me3 antibody-treated cell. **c** H3K9me3 Ab-GNP treated cell. 20 points were collected and averaged in each case. For comparison, full width half maximum (FWHM) is indicated.

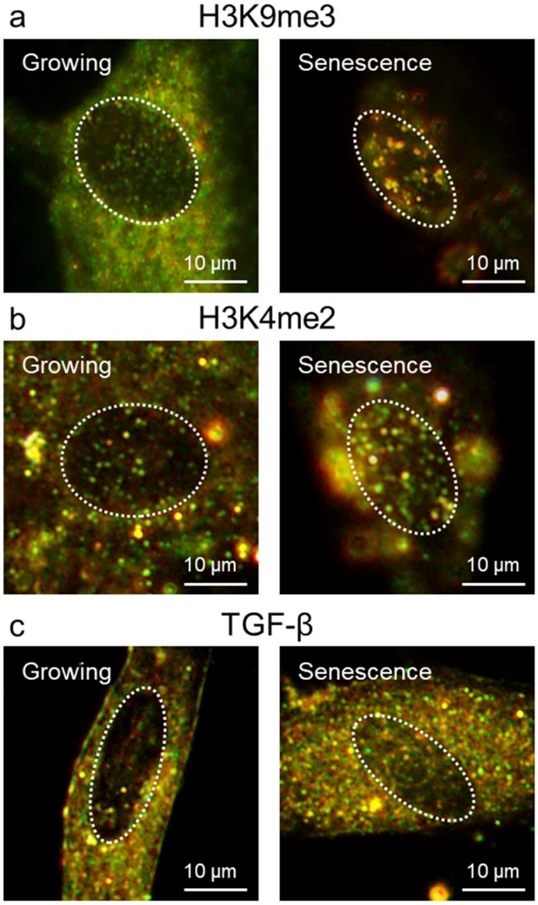

Supplementary Fig. 5 Dark-field scattering images of single cells using three different antibody-conjugated gold nanoprobes. **a** Cells treated with H3K9me3 antibody-conjugated gold nanoprobes. **b** Cells treated with H3K4me2 antibody-conjugated gold nanoprobes. **c** Cells treated with TGF-β antibody-conjugated gold nanoprobes. The nanoprobes were treated to growing cells (0 h after 4-OHT treatment) and senescent cells (144 h after 4-OHT treatment), respectively. All scale bars represent 10 μm. All images represent 3 experiments.

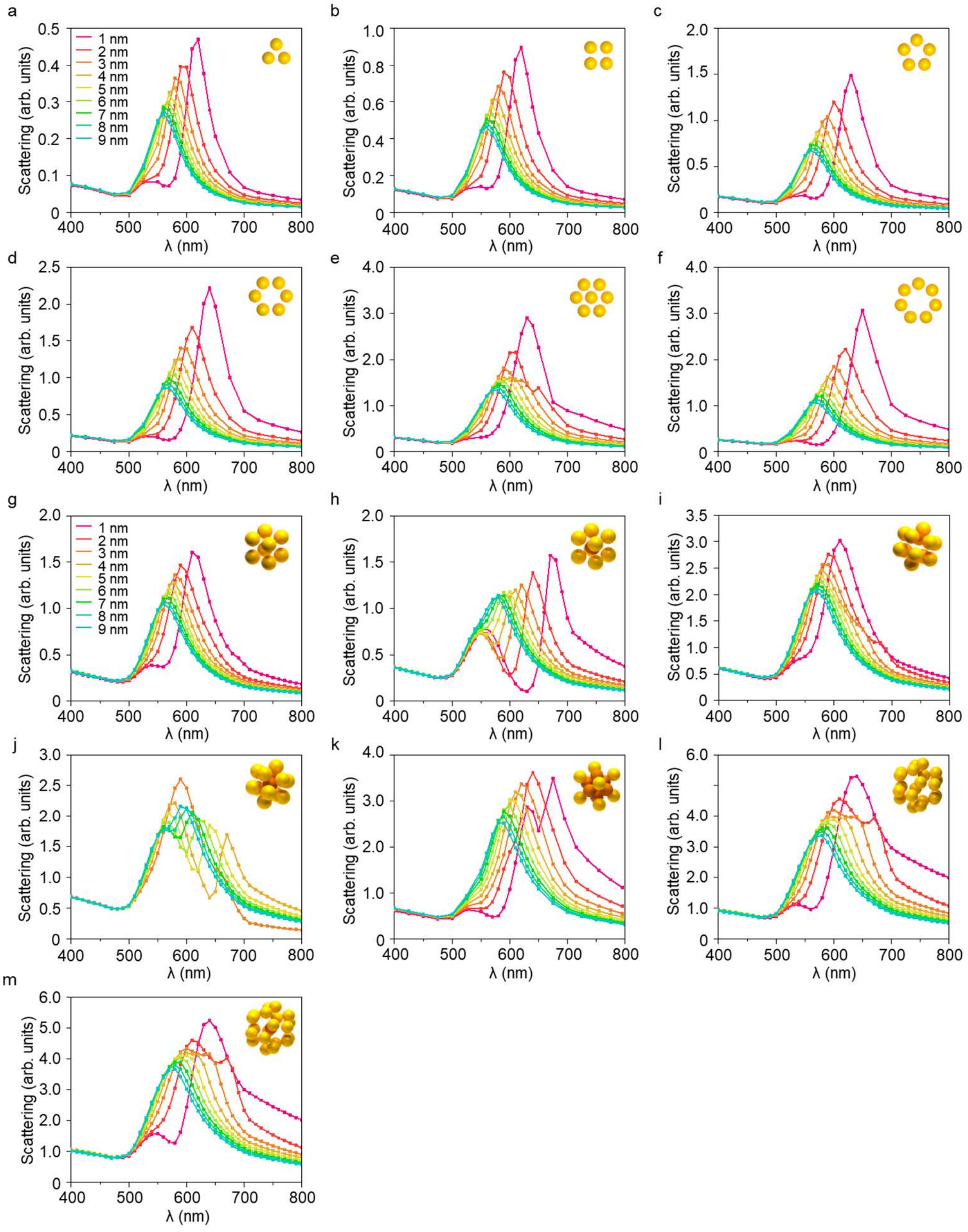

Supplementary Fig. 6 Computational simulations of the scattering spectra representing diverse optical properties according to the arranged GNPs in several 2D and 3D configurations. 2D arrangements: **a** trimer, **b** tetramer, **c**

pentamer, **d** hexamer, **e** hexamer with a core, and **f** heptamer. 3D arrangements: **g** octamer, **h** nonamer, **i** dodecamer, **j** dodecamer with a core, **k** tetradecamer, and **l** icosamer, **m** icosamer with a core.

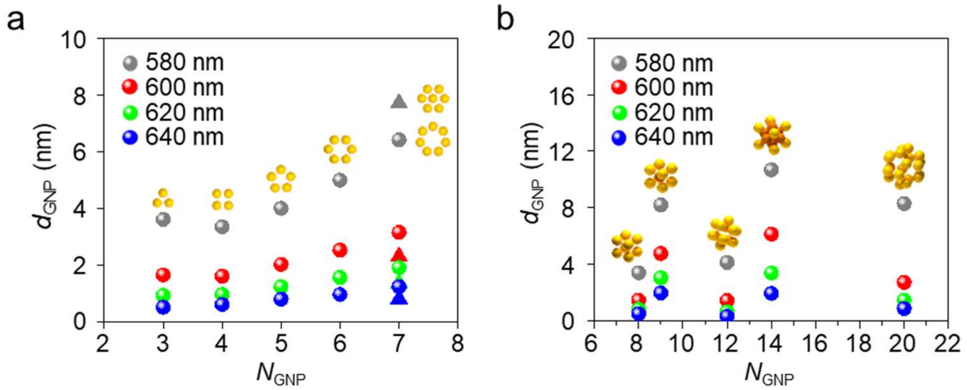

Supplementary Fig. 7 The expected interparticle distance for each λ_{max} according to the N_{GNP}. **a** 2D arrangements. The hexamer* and heptamer with the same N_{GNP} are presented with triangles and circles to distinguish them. **b** 3D arrangements. Corresponding 2D and 3D arrangements of the GNPs are depicted as inset.

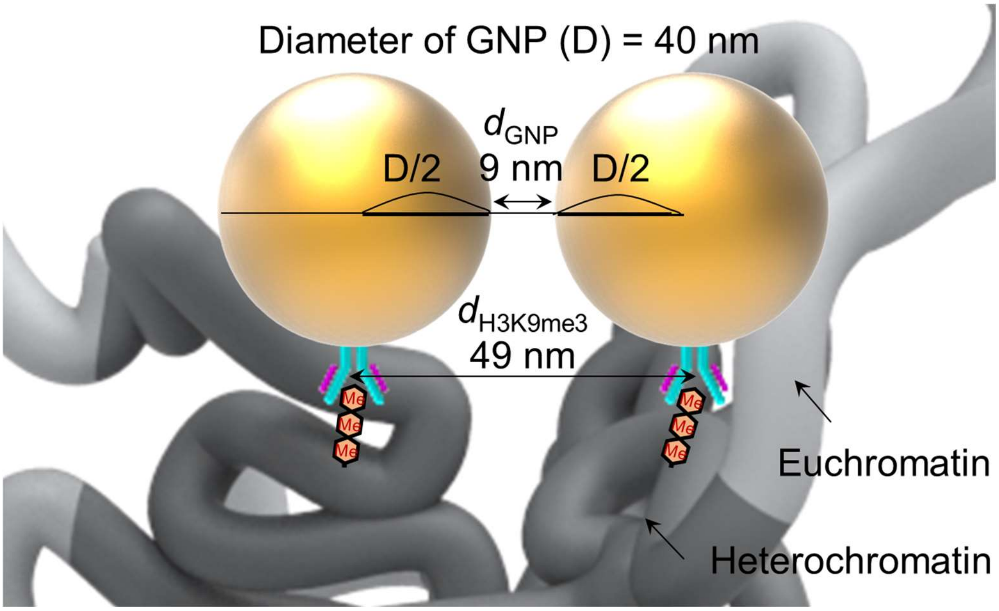

Supplementary Fig. 8 A schematic illustration of the estimation of the distance between histone markers on the basis of the numerical results of d_{GNP}, assuming that all Ab-GNPs attached in the same direction.

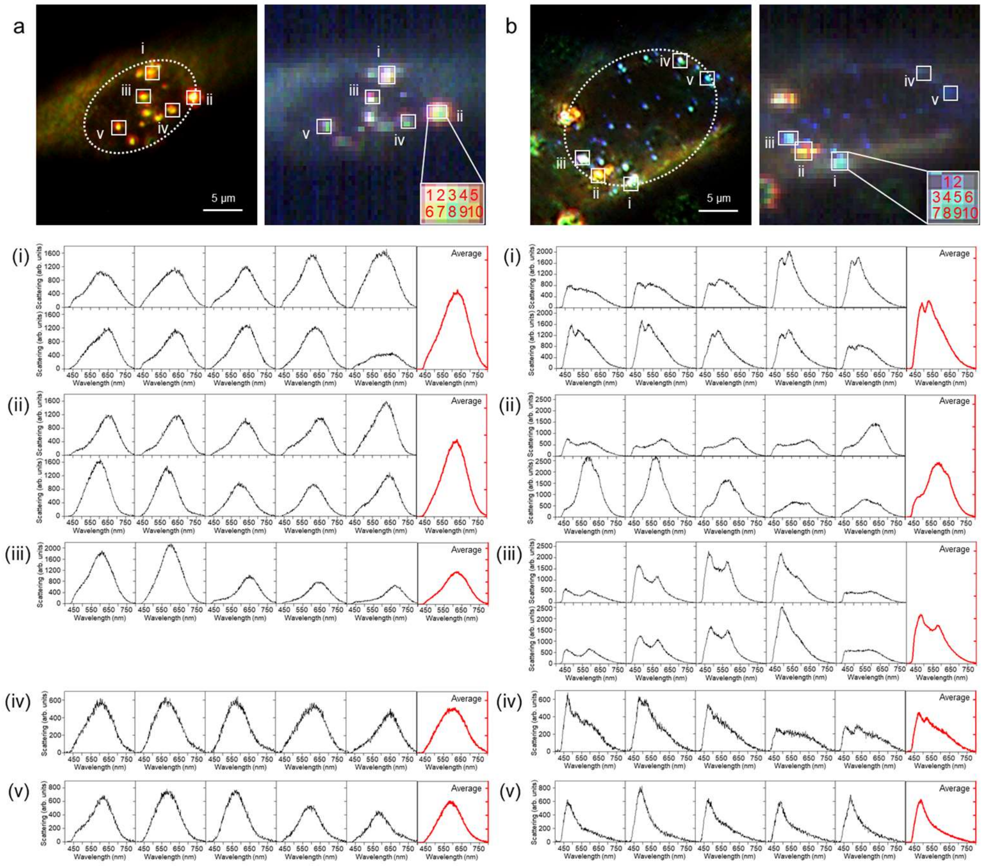

Supplementary Fig. 9 Scattering analyses of dual histone marker targeting. **a** Scattering (top left) and hyperspectral (top right) images of the OIS cell using only GNPs for two different histone markers (H3K9me3 and H3K27me3). Scale bar, 5 µm. **b** Scattering (top left) and hyperspectral (top right) images of the OIS cell using GNP for H3K9me3 and SNP for H3K27me3. Scale bar, 5 µm. (i–v) Representative scattering spectra measured from the observed scattering spots. Individual spectra collected from each pixel in the single

scattering spot are shown in black. An average of the collected spectra is shown in red.

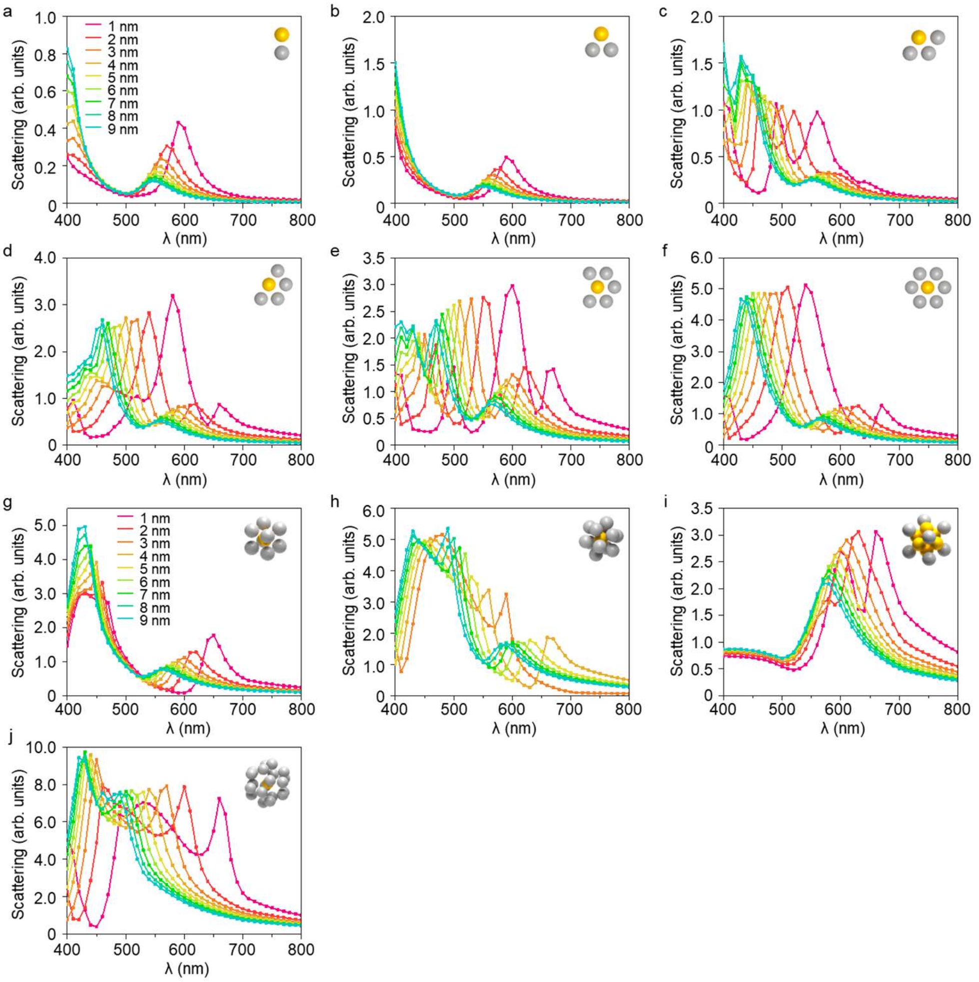

Supplementary Fig. 10 Computational simulations of the scattering spectra representing diverse optical properties according to several 2D (**a-f**) and 3D (**g-j**) arrangements consisting of GNPs (core) and SNPs (satellites).

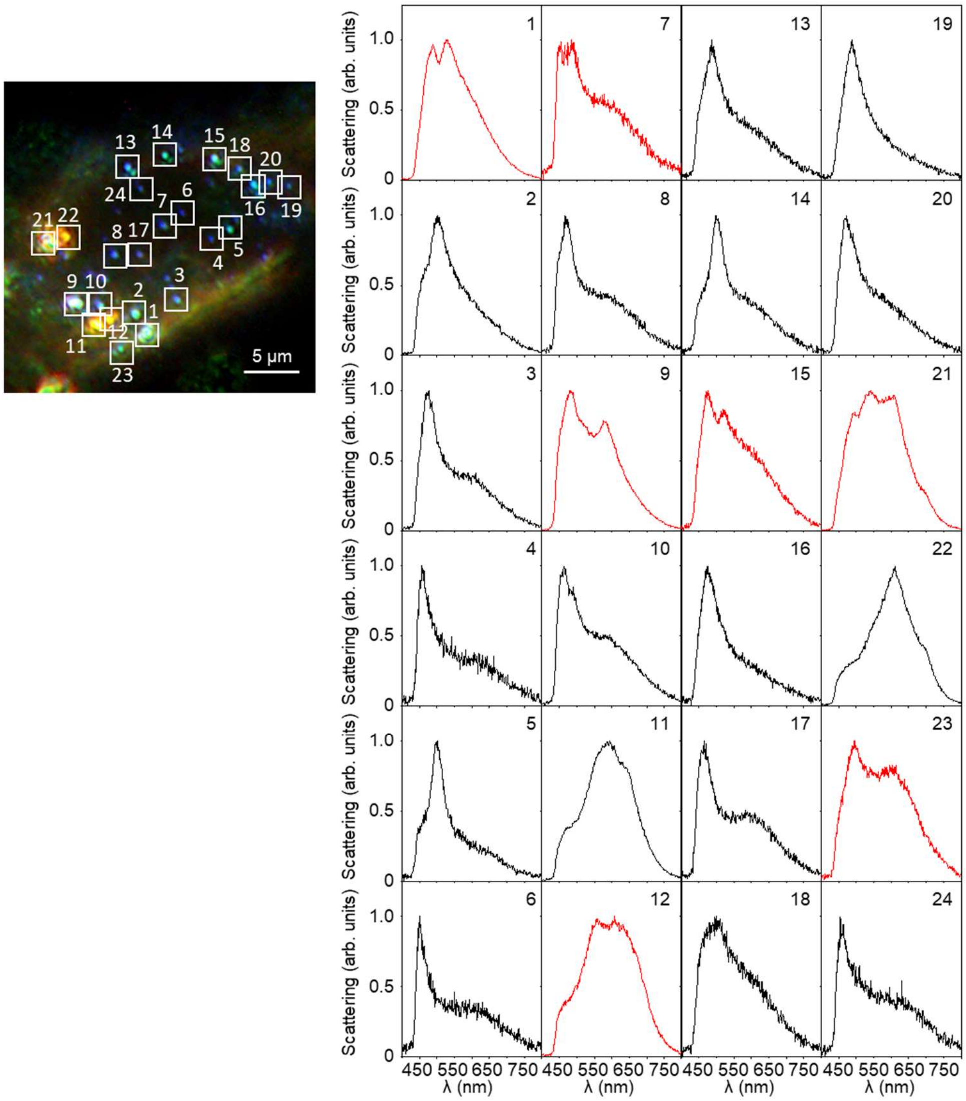

Supplementary Fig. 11 Representative scattering spectra collected from the scattering spots observed in single nucleus targeting with GNP for H3K9me3 and SNP for H3K27me3. Red graphs indicate spectra including multiple peaks. Scale bar, 5 μm.
