# Supplementary material for: High-spatial and colourimetric imaging of histone modifications in single senescent cells using plasmonic nanoprobes
Source: Nat Commun. 2021 Oct 8;12:5899. doi: 10.1038/s41467-021-26224-9 (PMC8501099; doi:10.1038/s41467-021-26224-9)
Supplement: Supplementary file 2 — Reporting Summary [file 41467_2021_26224_MOESM2_ESM.pdf]

nature portfolio

Corresponding author(s): Inhee Choi

Last updated by author(s): Aug 16, 2021

Reporting Summary

Nature Portfolio wishes to improve the reproducibility of the work that we publish. This form provides structure for consistency and transparency in reporting. For further information on Nature Portfolio policies, see our Editorial Policies and the Editorial Policy Checklist.

Statistics

For all statistical analyses, confirm that the following items are present in the figure legend, table legend, main text, or Methods section.

n/a	Confirmed	
☐	☒	The exact sample size (n) for each experimental group/condition, given as a discrete number and unit of measurement
☐	☒	A statement on whether measurements were taken from distinct samples or whether the same sample was measured repeatedly
☒	☐	The statistical test(s) used AND whether they are one- or two-sided<br>*Only common tests should be described solely by name; describe more complex techniques in the Methods section.*
☒	☐	A description of all covariates tested
☒	☐	A description of any assumptions or corrections, such as tests of normality and adjustment for multiple comparisons
☐	☒	A full description of the statistical parameters including central tendency (e.g. means) or other basic estimates (e.g. regression coefficient) AND variation (e.g. standard deviation) or associated estimates of uncertainty (e.g. confidence intervals)
☒	☐	For null hypothesis testing, the test statistic (e.g. F, t, r) with confidence intervals, effect sizes, degrees of freedom and P value noted<br>*Give P values as exact values whenever suitable.*
☒	☐	For Bayesian analysis, information on the choice of priors and Markov chain Monte Carlo settings
☒	☐	For hierarchical and complex designs, identification of the appropriate level for tests and full reporting of outcomes
☒	☐	Estimates of effect sizes (e.g. Cohen's d, Pearson's r), indicating how they were calculated

Our web collection on statistics for biologists contains articles on many of the points above.

Software and code

Policy information about availability of computer code

Data collection	All simulations were carried out using commercial software named COMSOL Multiphysics 5.4.
Data analysis	All simulations were carried out using commercial software named COMSOL Multiphysics 5.4.

For manuscripts utilizing custom algorithms or software that are central to the research but not yet described in published literature, software must be made available to editors and reviewers. We strongly encourage code deposition in a community repository (e.g. GitHub). See the Nature Portfolio guidelines for submitting code & software for further information.

Data

Policy information about availability of data

All manuscripts must include a data availability statement. This statement should provide the following information, where applicable:

- Accession codes, unique identifiers, or web links for publicly available datasets
- A description of any restrictions on data availability
- For clinical datasets or third party data, please ensure that the statement adheres to our policy

Data that support the findings of this study are available in Figshare with the identifier doi: 10.6084/m9.figshare.15169914.

Field-specific reporting

Please select the one below that is the best fit for your research. If you are not sure, read the appropriate sections before making your selection.

☒ Life sciences ☐ Behavioural & social sciences ☐ Ecological, evolutionary & environmental sciences

For a reference copy of the document with all sections, see nature.com/documents/nr-reporting-summary-flat.pdf

Life sciences study design

All studies must disclose on these points even when the disclosure is negative.

Sample size	We determined the sample size as 50 spots in each cell, which is also the minimum number of the spots that can be observed as senescence-associated heterochromatin foci (SAHF) in the nucleus of 144-hour OIS cell. Also, we conducted 5 independently repeated experiments and determined 15 cells as a sample size to show data reliability.
Data exclusions	The data with exceptional spectra shape or non-stained cells were excluded from the analyses. Since the plasmonic nanoprobes normally showed sharp-shaped spectra and the cellular organelles showed broad-shaped spectra, we pre-established the exclusion criteria.
Replication	To verify the reproducibility, we repeated the experiments more than 5 times and obtained high reproducibility except a few cases. In the non-replicated cases, nuclear membrane was not sufficiently permeabilized or plasmonic nanoprobes were pre-aggregated before entering into the nucleus
Randomization	In our typical experiment, we repeatedly incubated cells with different OIS time and fixed the incubated cells to multiple glass slides. Then, we randomly selected the cells from the slide glass for imaging cells at each time point of OIS.
Blinding	Our study aims to first show the correlation of the analyzing results using new imaging probe (plasmonic probe) with OIS time point. Thus, we did not use blinded samples. To this purpose, the OIS cell samples were provided by collaborative researchers with labeling the OIS time.

Reporting for specific materials, systems and methods

We require information from authors about some types of materials, experimental systems and methods used in many studies. Here, indicate whether each material, system or method listed is relevant to your study. If you are not sure if a list item applies to your research, read the appropriate section before selecting a response.

Materials & experimental systems

n/a	Involved in the study	
☐	☒	Antibodies
☐	☒	Eukaryotic cell lines
☒	☐	Palaeontology and archaeology
☒	☐	Animals and other organisms
☒	☐	Human research participants
☒	☐	Clinical data
☒	☐	Dual use research of concern

Methods

n/a	Involved in the study	
☒	☐	ChIP-seq
☒	☐	Flow cytometry
☒	☐	MRI-based neuroimaging

Antibodies

Antibodies used	<Primary antibodies><br>1. Anti-Histone H3 (tri methyl K9) antibody, Abcam, Cat. No. ab8898<br>2. Anti-Histone H3 (tri methyl K27) antibody, Millipore, Cat. No. 07-449<br>3. Anti-Histone H3 (di methyl K4) antibody, Abcam, Cat. No. ab7766<br>4. TGF-β1 polyclonal antibody, Biorbyt, Cat. No. orb114379<br><br><Secondary antibodies><br>5. Goat anti-Rabbit IgG (H+L) Cross-Adsorbed Secondary Antibody, Alexa Fluor 488, Invitrogen, Cat. No. A-11008
Validation	1. Anti-Histone H3 (tri methyl K9) antibody<br>: Host- Rabbit<br>Suitable for: WB, IHC-P, ICC, ChIP<br>Species Reactivity: Mouse, Cow, Human<br><br>2. Anti-Histone H3 (tri methyl K27) antibody<br>: Host- Rabbit<br>Suitable for: ICC, IP, Mplex, WB, IHC<br>Species Reactivity: Mouse, Human

3. Anti-Histone H3 (di methyl K4) antibody
: Host- Rabbit
Suitable for: ICC, ChIP, WB, PepArr
Species Reactivity: Mouse, Rat, Cow, Human

4. TGF-β1 polyclonal antibody
: Host- Rabbit
Suitable for: ELISA, IF, IHC-P, WB
Species Reactivity: Mouse, Rat, Human

5. Goat anti-Rabbit IgG (H+L) Cross-Adsorbed Secondary Antibody, Alexa Fluor 488
: Host- Goat
Species Reactivity: Rabbit
Conjugate: Alexa Fluor® 488
Cross Adsorption: Against human IgG, human serum, mouse IgG, mouse serum and bovine serum

Eukaryotic cell lines

Policy information about cell lines

Cell line source(s)	Human lung fibroblast IMR-90 cells were obtained from the ATCC (#CCL-186).
Authentication	Cell identity was confirmed by ATCC using STR (short tandem repeats) genotyping.
Mycoplasma contamination	IMR-90 cells used in this study were tested negative for mycoplasma contamination.
Commonly misidentified lines (See ICLAC register)	No commonly misidentified cell lines were used.
